# Visualizing intratumoral injections in lung tumors by endobronchial ultrasound

**DOI:** 10.7150/jca.81793

**Published:** 2023-02-22

**Authors:** Vitor Mori, Emily A. DuComb, Sarah Wagner, Farrah Khan, Jason H. T. Bates, C. Matthew Kinsey

**Affiliations:** 1 Vermont Lung Center, Pulmonary and Critical Care Medicine, Department of Medicine, University of Vermont College of Medicine, Burlington, VT 05405, USA.; 2 Division of Hematology and Oncology, Department of Medicine, University of Vermont, Burlington VT 05405.

**Keywords:** EBUS-TBNI, Intratumoral Chemotherapy, Drug Dynamics

## Abstract

Real-time endobronchial ultrasound images are crucial for the accurate placement of the needle in peribronchial lung tumors and lymph nodes for diagnostic sampling. Beyond its role as a diagnostic tool, ultrasound-guided bronchoscopy can also aid the delivery of anti-cancer agents intratumorally, enabling diagnosis, staging, and treatment to occur within the same anesthesia, reducing the patient's burden. However, determining drug retention and distribution *in situ* remains challenging, albeit pivotal in assessing the success or failure of the therapeutic intervention. We hypothesized that ultrasound images acquired by the bronchoscope during the injection can provide qualitative and quantitative real-time information about drug transport. As a proof-of-concept, we retrospectively analyzed 13 videos of intratumoral cisplatin injections in advanced non-small cell lung cancers. We identified the injection and performed quantitative analysis through image processing and segmentation algorithms and mathematical models in 5 of them. We were able to infer the unlikeliness of a laminar flow through interstitial pores in favor of the emergence of tissue fractures. These data imply that the structural integrity of the tumor is a critical determinant of the ultimate distribution of an intratumorally delivered agent.

## Introduction

Ultrasound-guided bronchoscopy is a standard-of-care diagnostic tool in thoracic oncology that is widely used for accessing lung tumors and lymph nodes. Recently, this tool has also been used to guide the intratumoral injection of anti-cancer agents as a salvage therapy for recurrent centrally-located non-small cell lung cancer (NSCLC) 1. This has obvious potential advantages in terms of achieving high drug concentrations at the site of malignancy while avoiding excessive doses to the rest of the body and may have particular importance for NSCLC immunotherapy by increasing the local production of neoantigens and potentiating the effect of systemic therapy 2. Nevertheless, the efficacy of intratumoral injection is highly dependent on the quantity of injected drug retained within and distributed throughout the tumor. However, visualizing the injection procedure in order to infer how the drug is physically accommodated within the tumor has proven challenging due to the need to add a "label", thus altering the size and potentially the charge of the drug or biologic 3.

In the present study, we evaluated the potential of the ultrasound probe attached to the tip of the bronchoscope to provide real-time visualization of the injection procedure, thereby allowing us to infer physical mechanisms of drug accommodation within the tumor. Endobronchial ultrasound (EBUS) provides real-time continuous imaging of peribronchial tumors in order to guide accurate needle placement for intratumoral therapy. Indeed, during clinical use of EBUS, the injection needle can be quite clearly seen as it enters the tumor. Furthermore, it is frequently possible to discern the ejection of the drug from the needle tip and its subsequent accumulation in the tumor tissue. We, therefore, surmised that it should be possible to assess drug retention and distribution within a tumor from a detailed analysis of the EBUS images obtained in patients undergoing intratumoral injection.

To address this question, we applied digital image analysis to routine clinical EBUS videos taken during the intratumoral injection of cisplatin in patients with advanced NSCLC. This analysis provided estimates of the fraction of injected drug retained within the field of view of the ultrasound image. We also used mathematical modeling methods to infer the physical mechanisms by which the retained drug was able to be accommodated by the tumor tissue.

## Materials and Methods

### Patient Data

This study was conducted in accordance with the Declaration of Helsinki (as revised in 2013) and was approved by the University of Vermont (UVM) Committee on Human Research in the Medical Sciences (CHRMS 17-075). All procedures were performed as clinically indicated. Informed consent was obtained for ancillary tissue and image analysis.

All ultrasound videos available from our database of cases were included, totaling 5 patients who had received EBUS-TBNI of cisplatin between May 2018 and March 2020 (Table 1). The cisplatin injections were given using a 19 G needle (Olympus, America, Center Valley, PA), and EBUS videos were recorded at 10 MHz with a curvilinear EBUS Bronchoscope (Olympus America, Center Valley, PA) attached to a Universal Ultrasound processor (EU-ME2, Olympus America, Center Valley, PA). The dose and injection strategy, i.e., number of injections and location(s), were selected by the treating physician based on tumor morphology, accessibility as determined by airway anatomy, and requirement to avoid large blood vessels. Details of this cohort have been previously published 4. In addition to the ultrasound image of the tumor and peribronchial structures, the EBUS scope simultaneously provides a white-light view of the airway. Following each injection, the needle track and surrounding airways were monitored for any backflow of the drug along the needle track or extravasation to the airways.

### Image Processing

**Figure 1:**
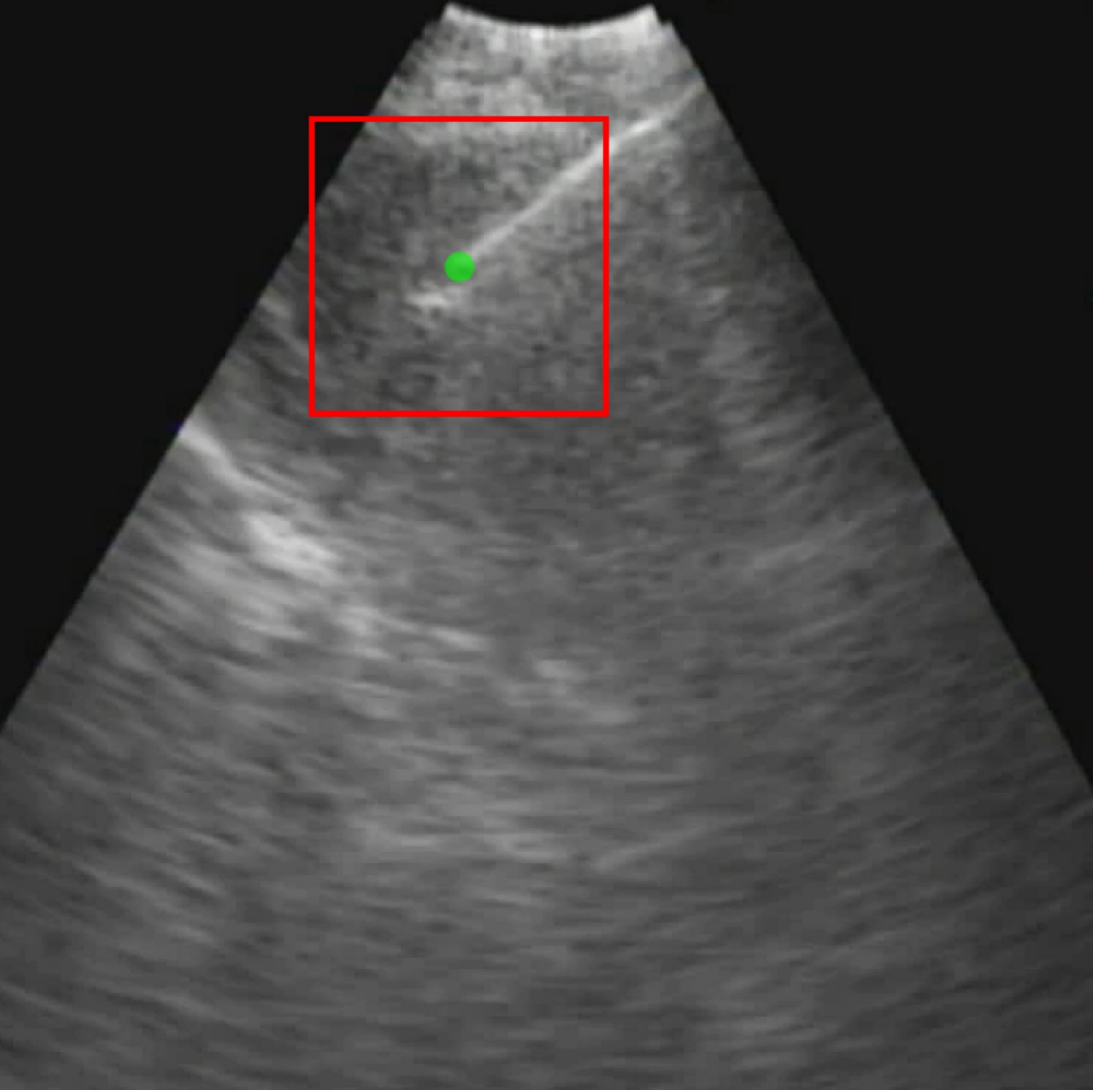
Initial frame from an EBUS image (B11). The green dot is the manually identified needle tip position centered within a 1 cm x 1 cm region of interest (red square). The tics along the right and bottom edges are separated by 5 mm.

Each video was analyzed within a mathematical programming environment with custom-designed software (MatLab 2020, The MathWorks, Natick, MA). The position of the needle tip in each video was manually identified, and a 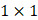 cm 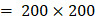 pixel region of interest (large enough to encompass the bolus of injected drug) was centered on the needle tip (Figure 1).

In a small subset of cases (2 out of 13 videos), the needle tip was not visible in the EBUS video, in which case we assumed the tip to be in the same position as for the previous injection within the same intervention for those who received multiple injections. For example, while the needle was not visible on A23, it was visible on A22. Therefore, the A23 needle tip position was assumed to be the same as for A22. The mean pixel intensity, 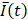, within the 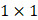 cm region of interest in each video frame was compared to the first frame of the video, 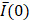, to yield a normalized percent intensity variation signal, 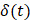, according to:


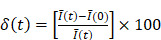

(1)

**Figure 2:**
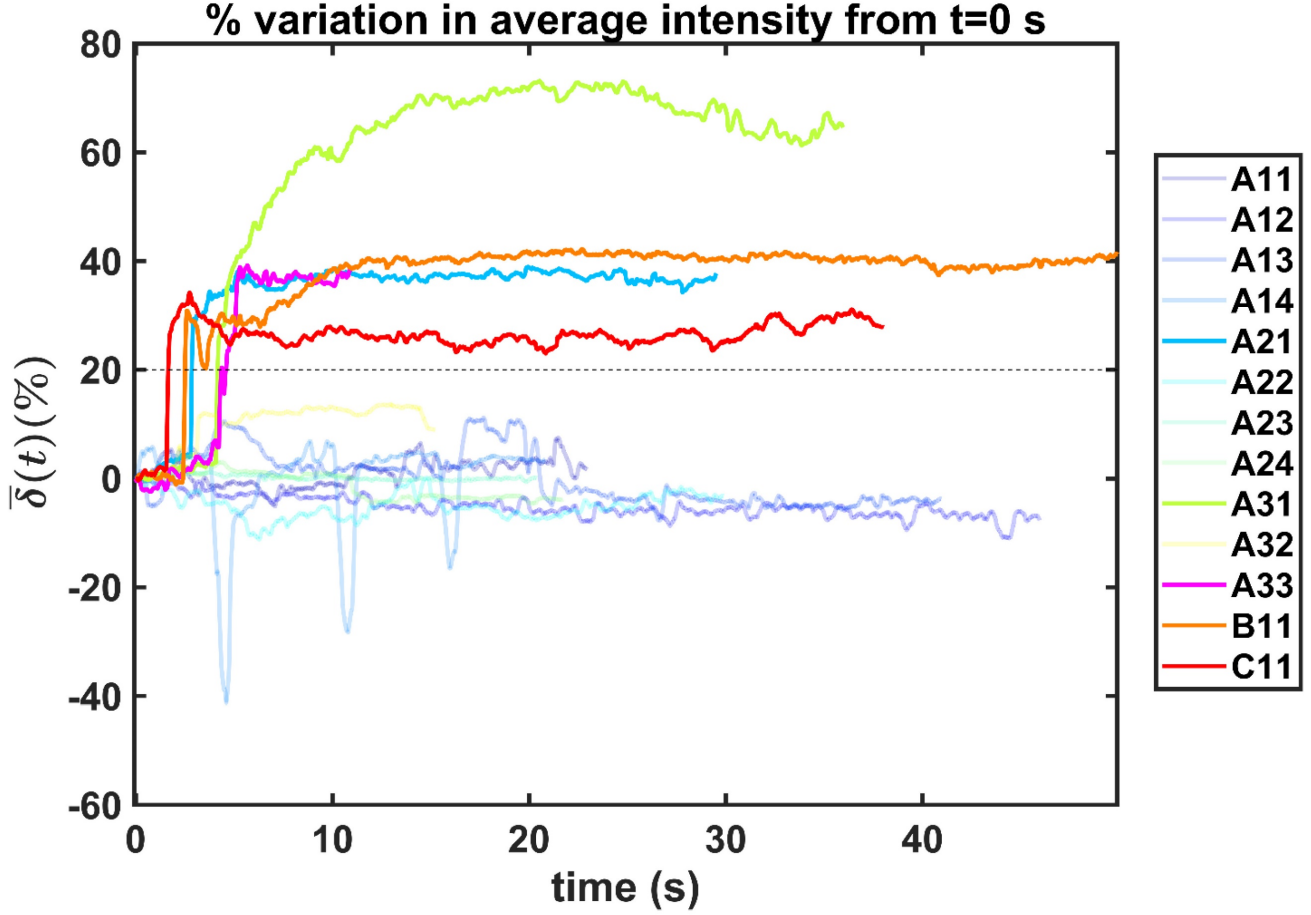
Percentual variation of the mean pixel intensity within the selected region of interest relative to the initial video frame.

In order to reduce noise artifacts generated by the heartbeat, 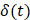 was smoothed using a 1-s moving average to yield 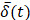. Only those cases for which drug dynamics following injection were clearly visible, defined as 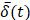 rising to a level above 20% (Figure 2), were retained for further analysis. This applied to subjects A21, A31, A33, B11, and C11 (Fig. 2).

**Figure 3:**
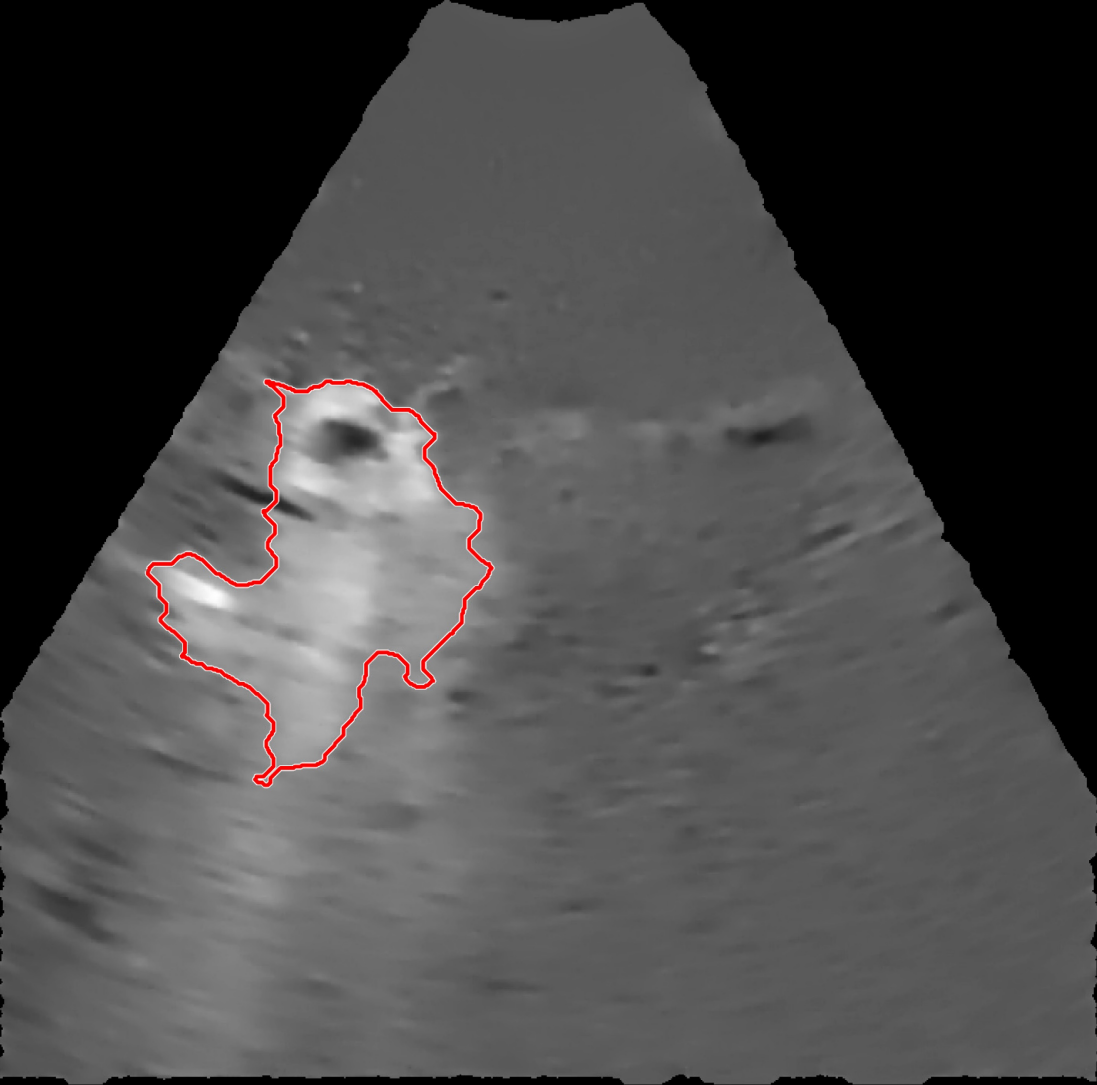
An example of a segmented region containing cisplatin superimposed on the filtered image.

The selected EBUS videos were then subjected to a series of image processing steps. First, the image background, defined as the mean of the images over the 1-s time window immediately preceding injection, was removed from all subsequent video frames. Second, a smoothing filter that enhanced edges by detecting areas with high-intensity gradients was applied to remove speckle noise without losing information concerning drug accumulation 5. Third, a threshold algorithm based on intensity clusters was applied for image segmentation 6. Lastly, morphological operations were applied to connect nearby segmented areas and fill inner holes while preserving the outer borders (see Supplementary Material for details). An example of a segmented area (red outline) corresponding to the drug depot within the ultrasound field of view superimposed on the filtered image with the background removed is shown in Figure 3.

Images of selected frames and videos are provided in the Supplementary Material.

### Image Analysis

Beginning at 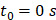, the time evolution of 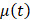, the average intensity in the EBUS image cone minus the average intensity of the background, was determined for each video and fit with the inverted exponential equation

*µ(t) = c+a1-e-bt* (2)

We defined the duration of the injection, 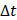, as the time interval between 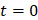 and the point at which 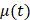 reached 95% of its asymptote value, given by


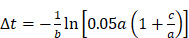

(3)

For simplicity, we assumed that the cisplatin flowed into the tumor during the injection at a constant rate, 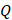, defined by the total injected volume divided by 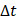.

We evaluated the time-evolution of the shape of the segmented region corresponding to the cisplatin in the tumor by imposing a 2-dimensional Cartesian coordinate system having its origin at the needle tip, its X-axis parallel to the needle axis, and its Y-axis perpendicular to the needle axis. We then calculated the paths of the centroids of the segmented area in both the X and Y directions, as well as the minimum and maximum Feret's diameters defined as the distances between the parallel lines that intersect the area boundaries at its minimum and maximum widths, respectively.

Lastly, we estimated the total amount of cisplatin retained in the tumor as the volume of rotation of the segmented area at the end of the injection. The difference between the total injected volume and this rotation volume then estimated the volume of drug escaping from the tumor during the injection.

### Modeling Physical Mechanisms of Drug Accommodation in the Tumor

Real-time visualization of drug injection and estimation of its spatio-temporal distribution can inform the likeliness of different physical mechanisms of intratumoral fluid transport and accommodation. For this analysis, we combined quantitative information from image analysis to mathematical models embracing two different mechanisms: 1) the laminar flow of a Newtonian fluid through a porous media, dominated by Darcy's law, and 2) a fracture initiated by the needle insertion propagating due to the high fluid pressure during the injection.

In order to assess the hypothesis that this movement was dominated by the flow of fluid through pores in the tumor tissue, we estimated the hydraulic conductivity of the tumor according to Darcy's law


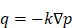

(4)

where 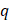 is the volumetric rate of drug flow per unit area 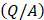, 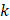 is the hydraulic conductivity, and 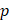 the pressure driving fluid flow within the tumor. Assuming a constant volumetric flow rate and radial symmetry around the needle tip, Equation 4 can be re-expressed in integral form as 7


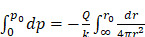

(5)

Assuming that 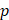 asymptotes to zero with distance from the needle and that fluid ceases to flow at the distance 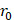 from the needle tip where 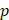 becomes equal to the tumor interstitial fluid pressure 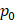, we obtain


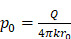

(6)

Another possibility is that the stress generated by the injections caused the tumor tissue to propagate the fracture generated by the needle insertion along which the cisplatin tunneled. According to Hutchinson and Suo 8, the threshold corresponds to the lowest stress 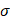 at which steady-state tunneling of a crack of width 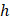 can occur in a layered material having elastic modulus 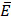 and mode I toughness 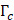 is


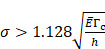

(7)

## Results

We did not observe backflow of cisplatin in the EBUS video images following injection in any of the patients, nor did we observe cisplatin extravasation to the surrounding airways. Nevertheless, there was a clear variation in drug distribution between the different subjects. For cases A21 and A31, the agent was distributed symmetrically around the Y-axis throughout the injection, during which the centroid moved away from the needle tip along the X-axis. In A33, the drug deflected to the right of the needle within 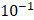 s from the start of the injection. In B11, the cisplatin spread around the tumor during the injection but accumulated at the tumor edges within a period of seconds. Lastly, in C11, the cisplatin distribution retained radial symmetry about the needle tip. Original and segmented videos are provided in the Supplementary Materials.

**Figure 4:**
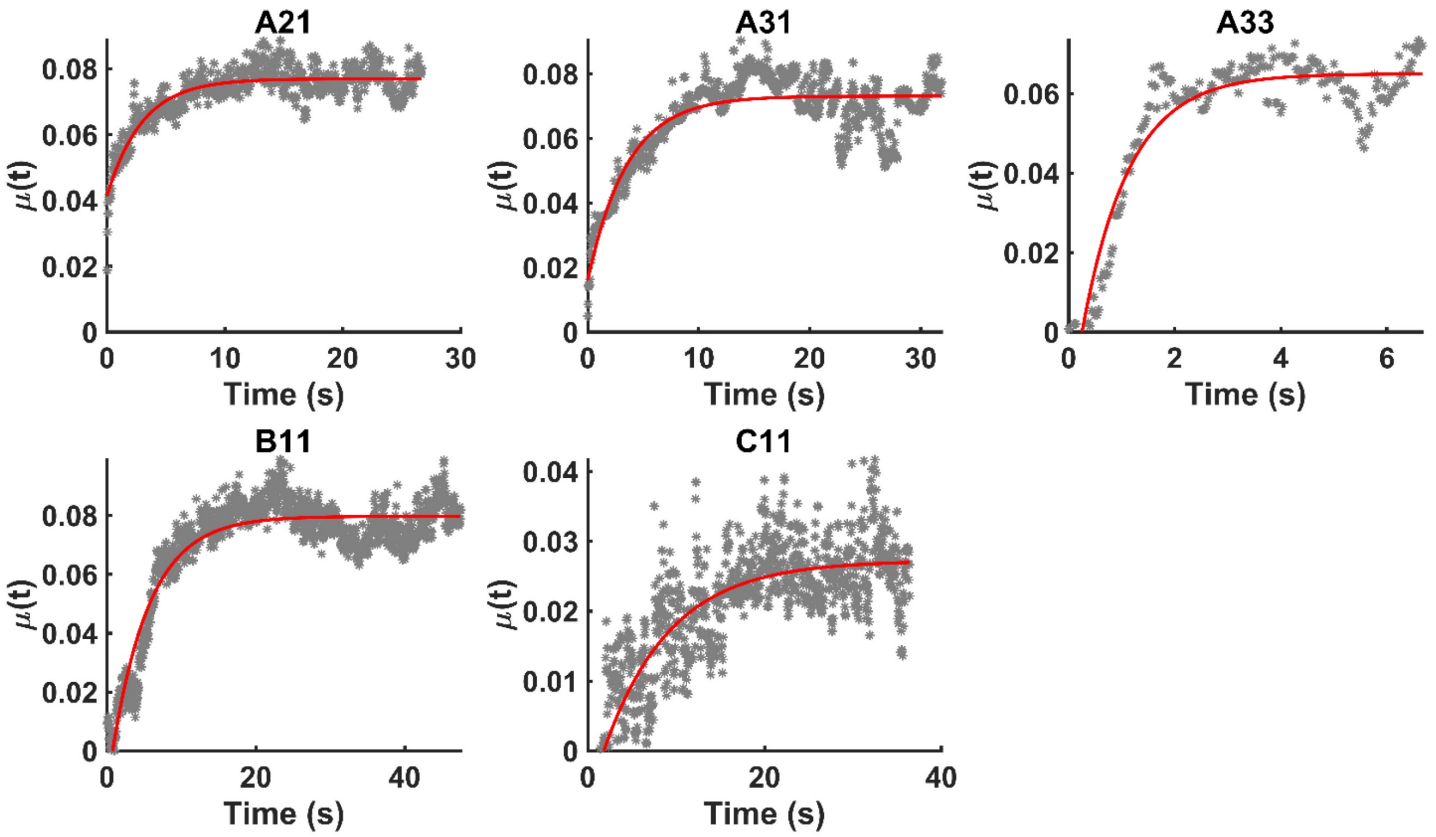
Time evolution of 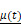 together with the model fits provided by Eq. 4 (red lines).

**Figure 5:**
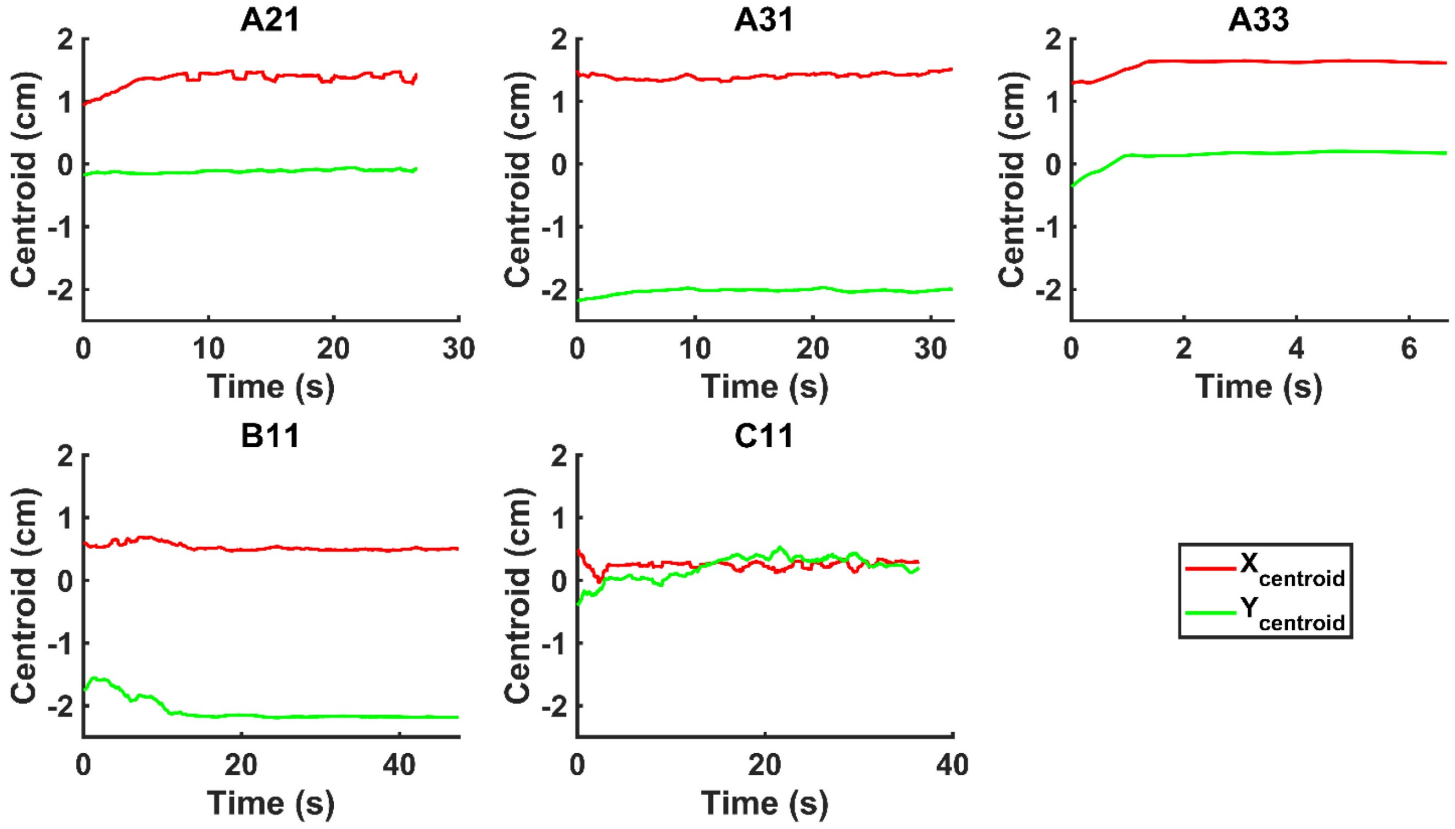
Time-evolution of the segmented area centroid in the X and Y directions.

**Figure 6:**
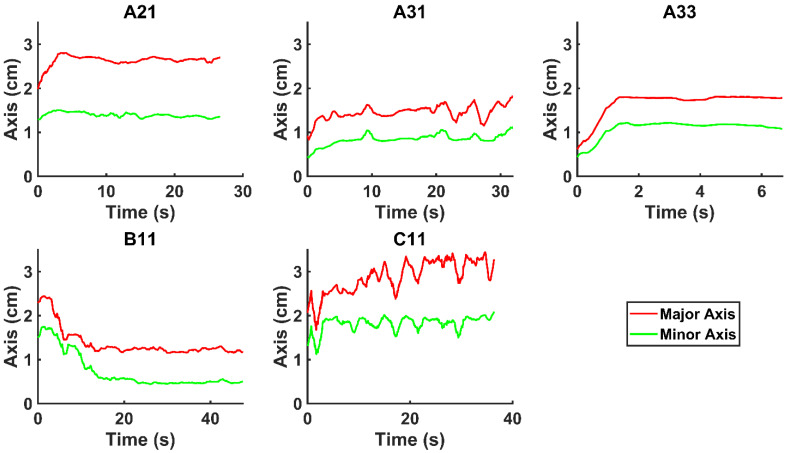
Time evolution of the minimum and maximum Feret's diameter of the segmented area.

**Figure 7:**
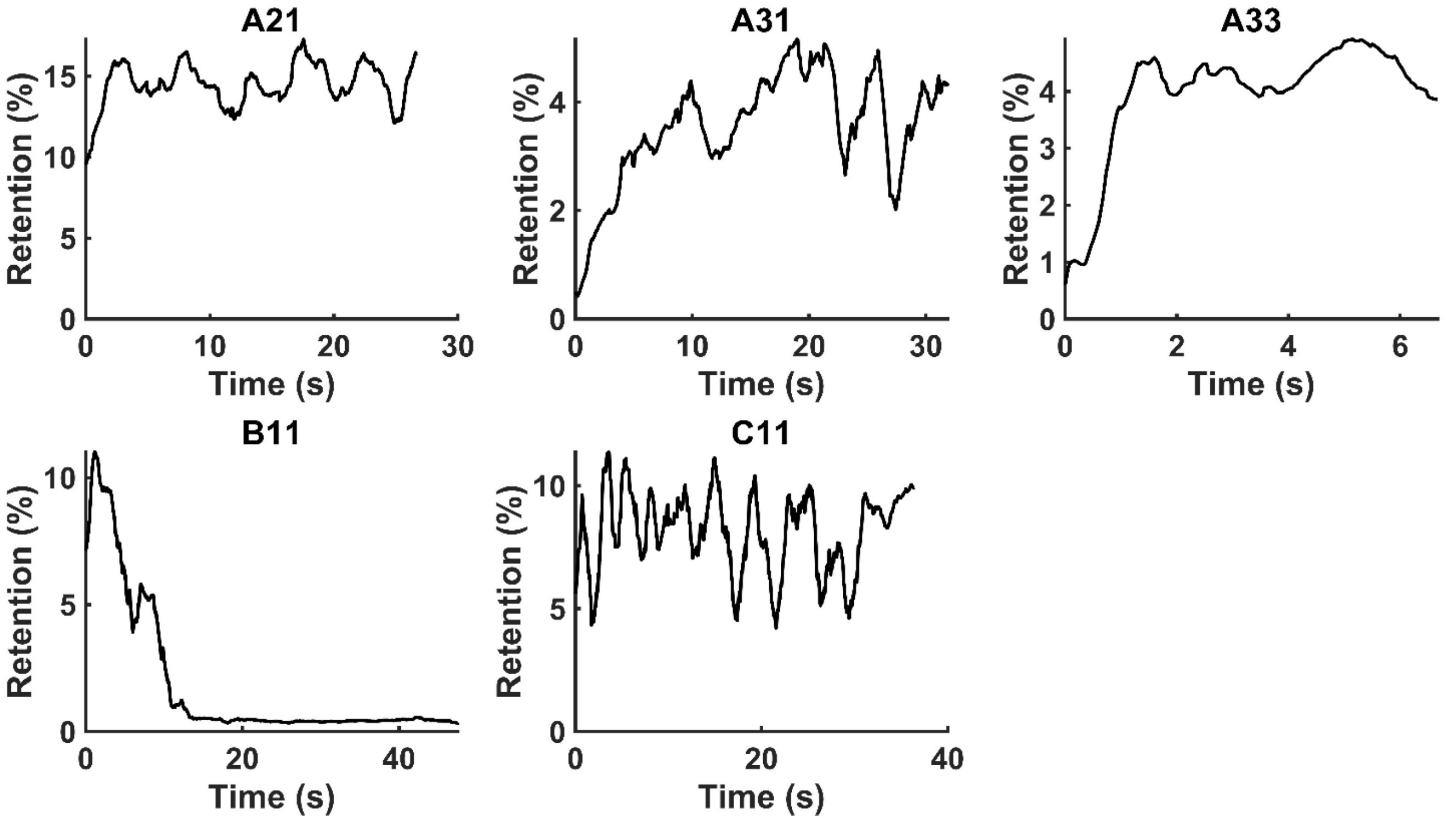
Moving averages (1-s window) of % drug retention in each tumor throughout the course of injection.

Figure 4 shows the time evolution of 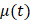 together with the fits provided by Equation 2. The estimated values of 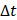 (Equation 3), 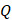 and 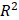 for the fits by Equation 4 are presented in Table 2 for each of the five injections. The time-evolutions of the segmented area centroid along the X and Y directions are shown in Figure 5. Figure 6 shows the maximum and minimum Feret's diameters of the segmented area as a function of time. Figure 7 presents the estimated % of injected drug retained in the tumors at the end of injection.

We used Eq. 6 to estimate the value of 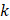 based on a value for 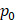 of 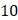 mmHg 9. Based on data from Figure 4 and Table 2, the injection flow rate 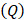 is set to the minimum limit value of 0.4 mL/s. Moreover, based on data from Figure 6, the estimated value for the depot size 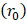 ranges between 0.1-1 cm. By applying them to Equation 6, combined with literature values for tumor interstitial fluid pressure, we estimated tumor hydraulic conductivity to range between 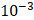 and 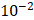 cm^2^.mmHg^-1^.s^-1^, higher than reported literature values of 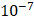 cm^2^.mmHg^-1^.s^-1^ 10, 11. Estimated and literature parameters for flow through porous media are summarized in Table 3.

We considered that the elastic modulus in lung tumors ranges from 20-30 kPa 12, 13. Assuming the limit scenario in which the segmented area in the ultrasound image represents a single contiguous macro-crackle, its width is around 1 cm, based on Figure 6. Assuming that fracture toughness in the order of magnitude of kJ/m2 as observed in adipose tissue 14 (4.1 kJ/m2) and porcine muscle 15 (2.5 kJ/m²), threshold stress for crack tunneling is around 125 kPa by applying Eq. 7.

Several different studies reported fluid pressure during intratumoral injections. Muñoz et al. 16 reported 30 kPa during intratumoral injection of an iodinated contrast agent using a 29 G needle at 50 µL/s. Assuming a linear relationship between pressure and flow rate, a 0.4 mL/s flow typical of our study would generate a pressure of around 240 kPa. Similarly, Comley and Fleck 7 found a linear relationship between flow from the needle (up to 0.1 mL/s) and needle tip pressure for PBS injected into subcutaneous porcine adipose tissue using 21 and 27 G needles. From their relationship, we calculated a needle tip pressure of 300 kPa for a 0.4 mL/s flow rate. Both estimations are higher than the threshold of 125 kPa calculated. Estimated and literature parameters for the emergence of a macro-crackle are summarized in Table 4.

We lastly assumed the possibility that several micro-crackles percolate and the low spatial resolution of the ultrasound image impaired the visualization of these fine structures. Micro-crack percolation will be modeled as fractures in brittle interfaces with a fracture toughness of 330 J/m2, as observed by Comley and Fleck in their study with adipose tissue 7. Moreover, we assumed an 800 µm wide crack width, following Comley and Fleck 7 observations for injections into adipose tissues. Again, the stress threshold was estimated to be around 125 kPa, while stress due to the injection was around 240-300 kPa. Parameter values are summarized in Table 5.

## Discussion

We have demonstrated that EBUS images acquired during the therapeutic injection of drug into a lung tumor can provide both qualitative and quantitative information about the manner in which the drug is accommodated by the tumor. This is a novel use of bronchoscopic ultrasound images. Importantly, this analysis utilizes data that are already collected as the standard of care and thus does not involve any additional risk to the patient. In the EBUS images in which we were able to discern the appearance of drug in the tumor tissue, our analysis indicates that the fraction of the injected drug retained within the EBUS field of view varied widely between patients. The amount retained, however, was below 20% in all cases, which is in accordance with values from *ex vivo* experiments 17. The remainder of the drug presumably escaped into the surrounding tissues and vasculature. Although this results in an order of magnitude improvement over systemic delivery, there is significant room for improvement in drug retention.

Backflow of drug through the channel in the tissue generated by the needle has been described as a major source of fluid leakage following injection 18. Nevertheless, we did not observe this phenomenon either in the white light optical images provided by the bronchoscope or in the EBUS videos. It is known that high-speed needle insertion and removal combined with the increased compressive stresses found in tumors can lead to the rapid closure of needle channels 19. This would limit backflow and may thus explain our observations. Other drug extravasation mechanisms, such as peritumoral leakage and vascular/lymphatic clearance, can also play a critical role in drug retention 20, but we were not able to observe these phenomena due to the low spatial resolution of EBUS images and/or the restricted field of view imposed by bronchoscope positioning.

We hypothesized that injected drug flowed through porous areas in the tumor space due to pressure gradients or induced the propagation of fractures in the tissue. Those propagated fractures could be a single major crack with the same dimension of the segmented area or the percolation of several micro-crackles, as previously observed by other studies 7, and this degree of granularity could not be resolved in ultrasound images. Based on image analysis and Eq. 6, we estimated tumor hydraulic conductivity to be 4 to 5 orders of magnitude greater than the values that have been previously reported for tumors 10, 11. Comley and Fleck 7 also found anomalously large values for 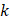 when studying injections in porcine adipose tissue. Since there is no reason to suspect extremely large values of 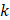 in the tumors we studied, it seems that Darcy flow through a porous medium was not a principal mechanism by which the drug was accommodated by the tumor.

Steady-state tunneling of a crack was proposed by Comley and Fleck 7 to explain the 800 µm wide micro-cracks they observed following injections into adipose tissues. Moreover, Morhard et al. 18 also observed fractures in an agarose surrogate for liver tissue with 100 and 200 µL injections at a flow of 10 mL/h, two orders of magnitude lower in both injectant volume and flow than in the present study. Indeed, estimations of the pressure generated by the injections with flow rates presented in Table 2 (240- 300 kPa) are higher than the stress threshold for fracture propagation, either considering the emergence of a single macro-crack or the percolation of multiple micro-cracks that occur in brittle interfaces. Although our results do not allow differentiating those two mechanisms, the high heterogeneity in the tumor mechanical microenvironment with known brittle areas such as necrotic, fibrotic, and vessel interfaces suggest that percolation of micro-cracks in fragile areas is a strong candidate to explain the distribution observed in the images.

However, it is also clear that there was some inter-subject variation in this regard. Fractures propagation directly away from the needle tip seems to have occurred in those patients for whom the drug was distributed symmetrically about the axis defined by the needle (A21, A33, and C11). On the other hand, in some patients (A31 and B11), the movement of the injectant was off-axis, which might have been due to crack propagation along brittle interfaces reflective of the particular tissue structure in those tumors. We also cannot discount the possibility that some small amount of Darcian flow occurred at the edges of the cavity created by tissue fracture.

The optimal scenario for realizing the therapeutic response to EBUS-TBNI delivery of a cytotoxic agent is an even drug distribution within the tumor without any leakage occurring through the tumor boundary or clearance through blood or lymphatic vessels. If the drug accumulates in a central macro-crack, dissemination to the rest of the tumor tissue will rely solely on diffusion, which is a slow process. Our previous modeling study 21 suggests that drug clearance from a tumor occurs at a much faster rate than drug diffusion within the tumor, thus decreasing the rate of drug retention when diffusion is relied on for distribution. Conversely, the percolation of micro-cracks facilitates the distribution of drug to the far reaches of the tumor but at the same time increases the risk of leakage through the tumor border due to bursting the containment capsule and can result in heterogeneous distribution due to accumulation in lower interstitial fluid pressure areas. Our previous work 21 also supports the use of a multiple injection strategy to better distribute drug throughout a tumor and abrogate this effect. The present study points to another potential advantage of multiple small injections namely reduced tissue disruption by limiting the amount of fracture produced 22, thereby reducing the risk of drug escaping from the tumor. Equation 6 shows that stress at the needle tip during injection increases the fracture risk, suggesting it may be beneficial to give the injection as slowly as possible. Indeed, Muñoz et al. 16 used a needle with multiple side holes to both reduce injection pressure and significantly enhance drug retention within the tumor. Intervening in the tumor mechanical microenvironment with strategies such as vascular normalization or stress alleviation 9 might also enhance intratumoral retention and distribution of injected agents. Finally, drug retention may be further improved by modifying the physical or chemical properties of the injectate, such as anchoring the active drug to carrier molecules 23, 24 or combining it with adjuvants that create synergistic local reactions in the tissue 25.

The present study has a number of limitations. Possibly most important of these is the small number of ultrasound videos that met our inclusion criteria for analysis. This limits our capacity to perform statistical tests and compare treatment efficacies, even though it still allows us to usefully assess possible physical mechanisms of drug accommodation within the tumor. We did not correlate our findings with clinical follow-up assessments of the patients. This is possible but is more severely limited by the small sample size. The usefulness of our analysis during clinical procedures would be enhanced by techniques that improve visualization of the drug delivery. Even for those cases that met the inclusion criteria in the present study, our analysis was limited by the low spatial resolution of the EBUS videos. While this resolution was sufficient for us to observe the general distribution of the injectate and thus to infer which were the dominant distribution mechanisms at play, it did not allow accurate quantification of the relative roles of these mechanisms. Despite these limitations, we were able to demonstrate for the first time that bronchoscopic ultrasound videos could be leveraged to infer mechanisms of drug distribution following intratumoral delivery.

In conclusion, we have shown that it is possible to extract quantitative information from clinical EBUS-TBNI ultrasound videos that reflect the distribution of cisplatin within a tumor immediately following injection. This may provide actional feedback that can be used by interventionalists to adjust their injection strategy during the procedure in order to improve drug retention and distribution. Analysis of these EBUS videos suggests that tissue fracture is the dominant mechanism by which drug is accommodated by a lung tumor, implying that the distribution dynamics of an intratumorally delivered agent are critically dependent on the structural integrity of the tumor tissue.

## Supplementary Material

Supplementary methods and figures.

Supplementary video A21.

Supplementary video A31.

Supplementary video A33.

Supplementary video B11.

Supplementary video C11.

## Figures and Tables

**Table 1:** Patient data

Patient	Intervention Date	Total dose (mg)	Number of Injections	Injection identification code
A	05/2018	32	4	A11, A12, A13, A14
A	04/2019	34	4	A21, A22, A23, A24
A	05/2019	30	3	A31, A32, A33
B	03/2020	20	1	B11
C	02/2020	20	1	C11

**Table 2:** Model parameters

	A21	A31	A33	B11	C11
Δt (s)	16.63	20.11	5.10	27.86	49.28
Q (mL/s)	0.48	0.42	1.96	0.72	0.41
R2	0.78	0.75	0.89	0.88	0.72

**Table 3:** Parameters estimations and literature values for flow through porous media

Flow through porous media: 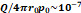 cm^2^.mmHg^-1^.s^-1^			
Parameter	Literature value	Estimated value	Reference
Injection flow rate 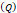	-	0.4 mL/s	-
Depot size 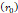	-	0.1-1 cm	-
Tumor interstitial fluid pressure 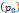	10 mmHg	-	9
Tumor hydraulic conductivity 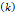	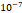 cm^2^.mmHg^-1^.s^-1^	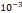 - 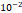 cm^2^.mmHg^-1^.s^-1^	10, 11

**Table 4:** Parameters estimations and literature values for the formation of a macro-crackle

Macro-crackle propagation: 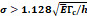			
Parameter	Literature value	Estimated value	Reference
Elastic modulus 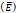	20-30 kPa	-	12, 13
Fracture toughness 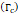	2.5-4.1 kJ/m^2^	-	14,15
Fracture width 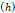	-	1 cm	-
Stress threshold ( 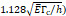	-	125 kPa	-
Stress 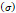	-	240-300 kPa	7,16

**Table 5:** Parameters estimations and literature values for the percolation of micro-crackles

Percolation of micro-crackles: 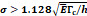			
Parameter	Literature value	Estimated value	Reference
Elastic modulus 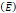	20-30 kPa	-	12, 13
Fracture toughness 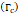	330 J/m^2^	-	7
Fracture width 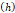	800 μm	-	7
Stress threshold ( 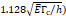	-	125 kPa	-
Stress 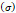	-	240-300 kPa	7,16
