# Supplementary material for: Visualizing intratumoral injections in lung tumors by endobronchial ultrasound
Source: J Cancer. 2023 Feb 22;14(4):544–53. doi: 10.7150/jca.81793 (PMC10088536; doi:10.7150/jca.81793)
Supplement: Supplementary file 1 — Supplementary methods and figures. [file jcav14p0544s1.pdf]

Visualizing intratumoral injections in lung tumors by endobronchial ultrasound

Vitor Mori[1*], Emily A. DuComb[1], Sarah Wagner[1], Farrah Khan[2], Jason H. T. Bates[1] and C. Matthew Kinsey[1]

[1]Vermont Lung Center, Pulmonary and Critical Care Medicine, Department of Medicine, University of Vermont College of Medicine, Burlington, VT 05405, USA

[2]Division of Hematology and Oncology, Department of Medicine, University of Vermont, Burlington VT 05405

Short title: **Ultrasound imaging of intratumoral injections**

*Corresponding Author:

Vitor Mori

149 Beaumont Ave, HSRF Room 233

Burlington, VT 05405

Tel: (802) 355-2485

Email: vitor.mori@uvm.edu

Image Processing and Segmentation Methodology

For the five videos that met the inclusion criteria, the frame corresponding to the start of injection was selected as that having the maximum time rate of change, $\dot{\bar{I}}(t_0) = \max\left[\frac{d\bar{I}(t)}{dt}\right]$. A video background image, B_{ij}, of the whole ultrasound area was calculated by averaging each pixel over a 1-s time window immediately prior to injection. B_{ij} was subtracted from all pixels, $I_{ij}(t)$, at each time point, t, in the video to yield

$$\hat{I}_{ij}(t) = I_{ij}(t) - B_{ij} \tag{2}$$

Next, a normalized video image, N_{ij}, was calculated as

$$N_{ij}(t) = \frac{\hat{I}_{ij}(t) - \min[\hat{I}_{ij}(t)]}{\max[\hat{I}_{ij}(t)] - \min[\hat{I}_{ij}(t)]} \tag{3}$$

We set the pixel intensity outside the ultrasound image cone to be zero. To perform edge-aware speckle noise reduction in the remainder of the ultrasound image, we applied a local Laplacian filter[2] to $N_{ij}(t)$ to produce the filtered video $\widehat{N}_{ij}(t)$. The parameters of the Laplacian filter were $\sigma = 0.2$ (amplitude of edges) and $\alpha = 3$ (smoothing of details in the input image while preserving edges).

Each frame of $\widehat{N}_{ij}(t)$ was segmented into four structures: 1) the dark background, 2) the ultrasound cone, $K_{ij}^{\alpha}(t)$, 3) bright structures in the background representing tumor edges and the needle that appears as a darker area in the filtered image due to background removal, $K_{ij}^{\beta}(t)$, and 4) the growing region of injected cisplatin at the needle tip, $K_{ij}^{\gamma}(t)$. This was achieved using three different threshold levels with the Otsu method[1], which relies on minimizing the intensity variance within each segmented region. The steps involved are illustrated in Figure S1.

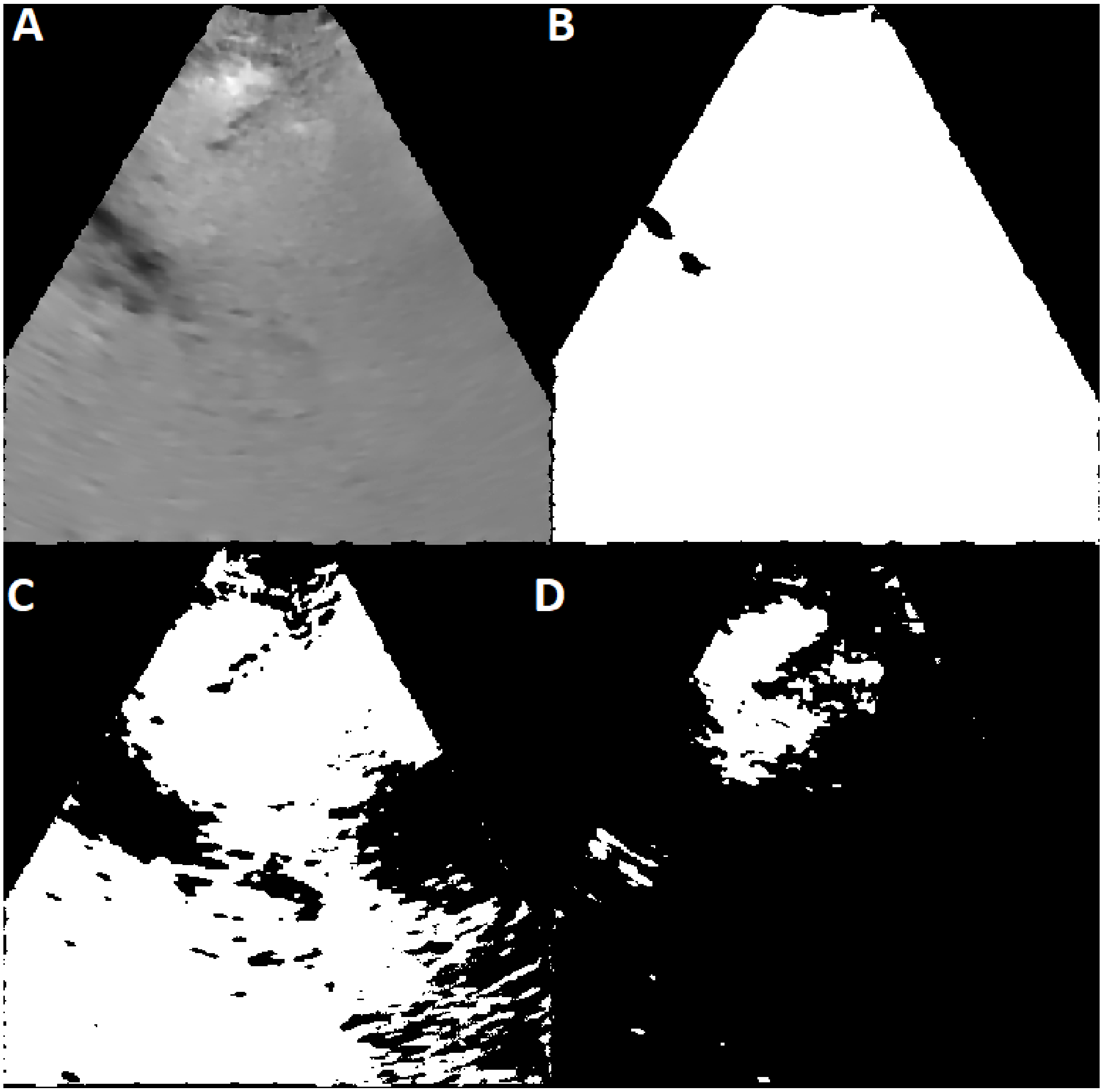

Figure S1: A) Filtered image without the background $\left(\widehat{N}_{ij}(t)\right)$ B) First threshold level, segmenting the ultrasound cone $\left(K_{ij}^{\alpha}(t)\right)$ C) Second threshold level, segmenting bright structures in the background (tumor edges and the needle) $\left(K_{ij}^{\beta}(t)\right)$ D) Third threshold level, segmenting the injected cisplatin in the tumor $\left(K_{ij}^{\gamma}(t)\right)$

Lastly, $K_{ij}^{\gamma}(t)$ was morphologically closed through a process of dilation and erosion. First, each point along the identified edge of $K_{ij}^{\gamma}(t)$ was dilated with 9-pixel octagon (structured element), further connecting white components whose structured elements overlapped. Then the structured element was removed from the edges of the resulting dilated image, eroding it. This preserved the outer borders of the segmented components, connecting those who were close enough. Next, we selected the white component with the largest area and

filled its inner holes. Finally, the selected segmented region was superimposed on the filtered image, $\widehat{N}_{ij}(t)$, to produce $P_{ij}(t)$, an example of which is shown in Figure S2.

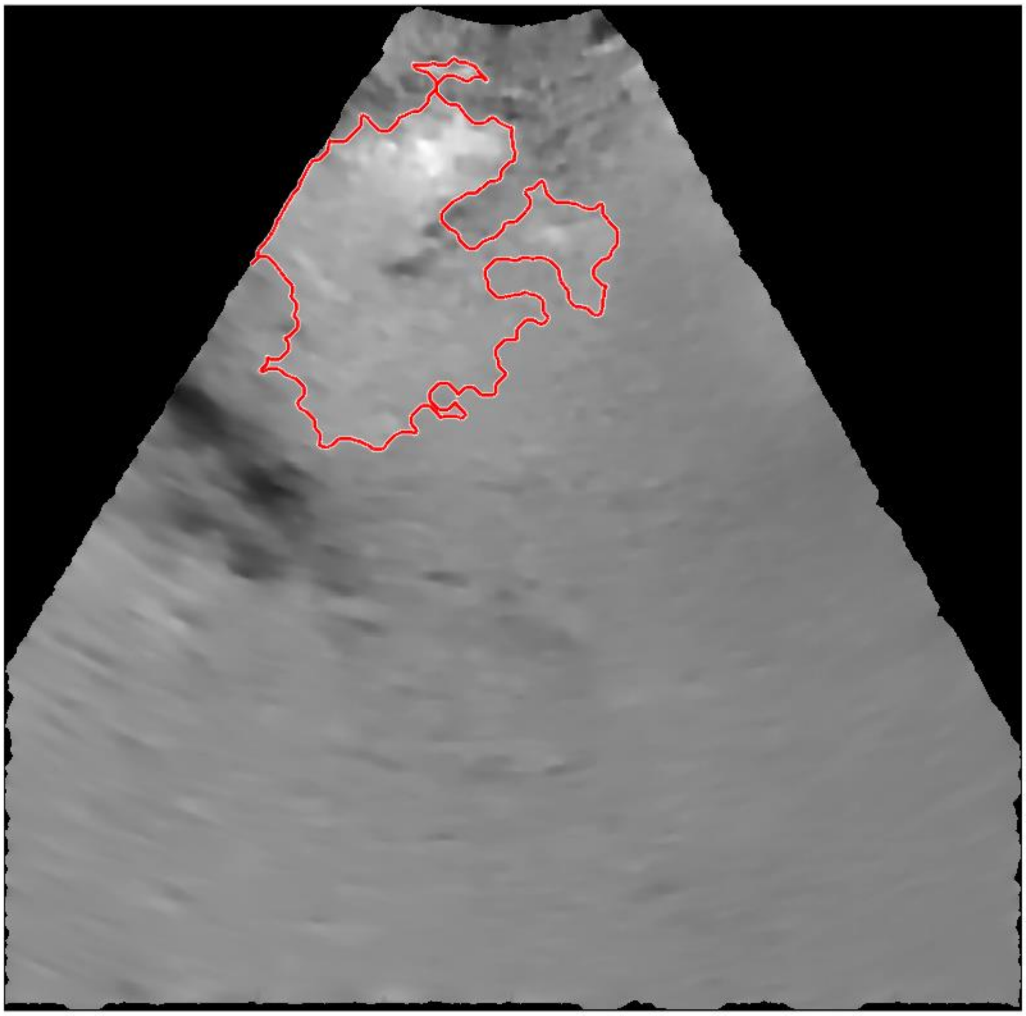

Figure S2: An example of $P_{ij}(t)$, consisting of a segmented region containing cisplatin superimposed on the filtered image.

Images of selected frames

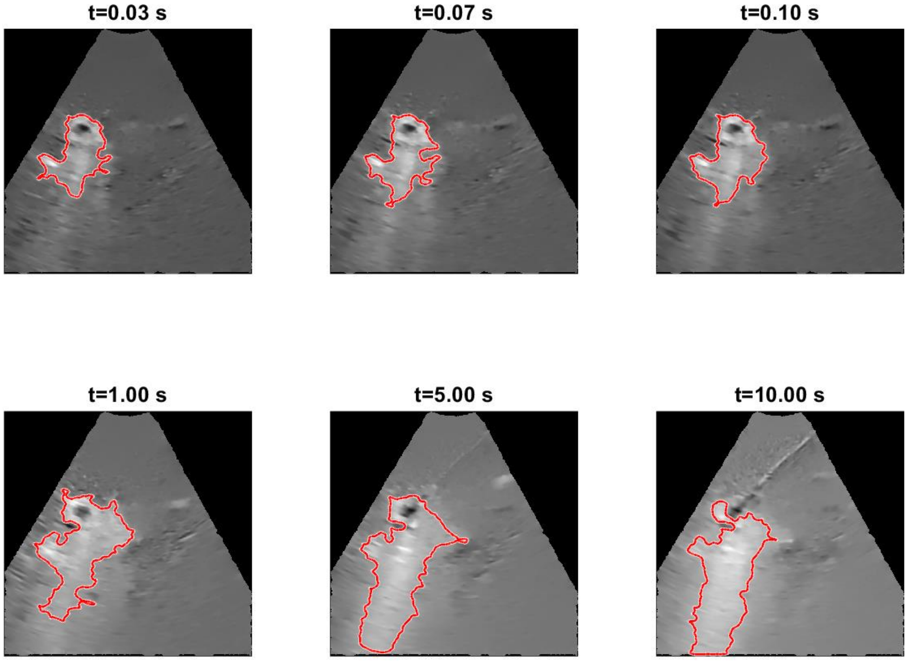

Figure S3: Selected frames of the filtered and segmented video for subject A21

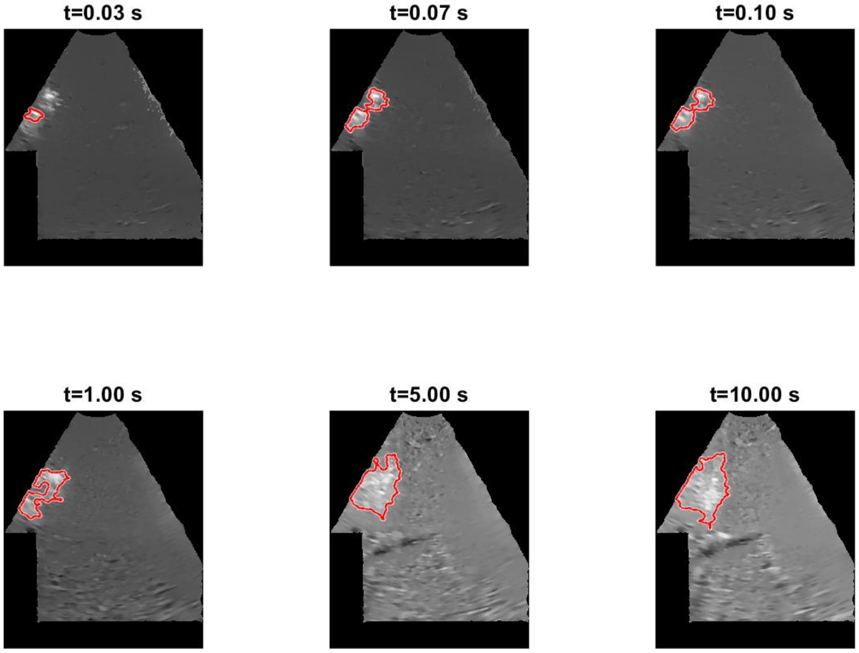

Figure S4: Selected frames of the filtered and segmented video for subject A31

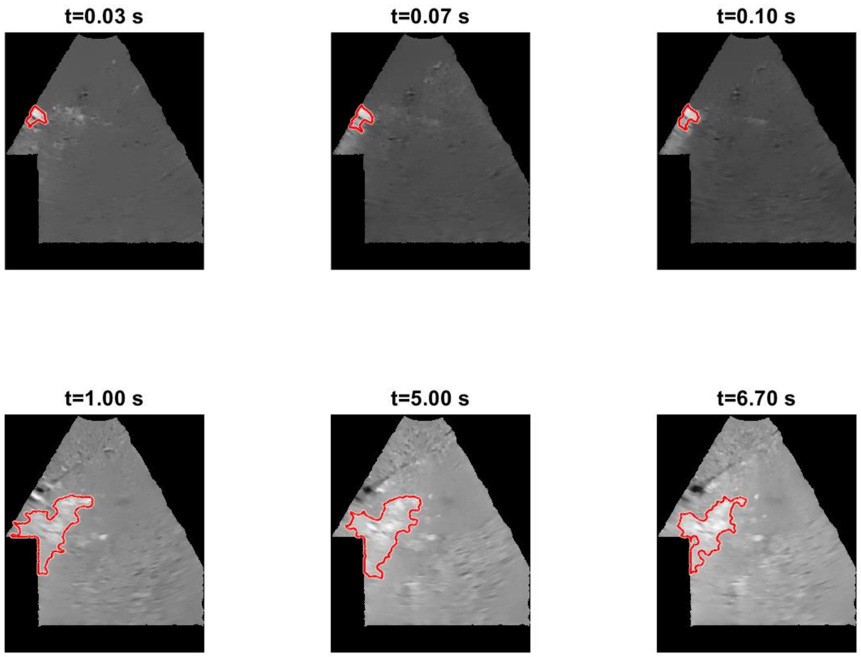

Figure S5: Selected frames of the filtered and segmented video for subject A33

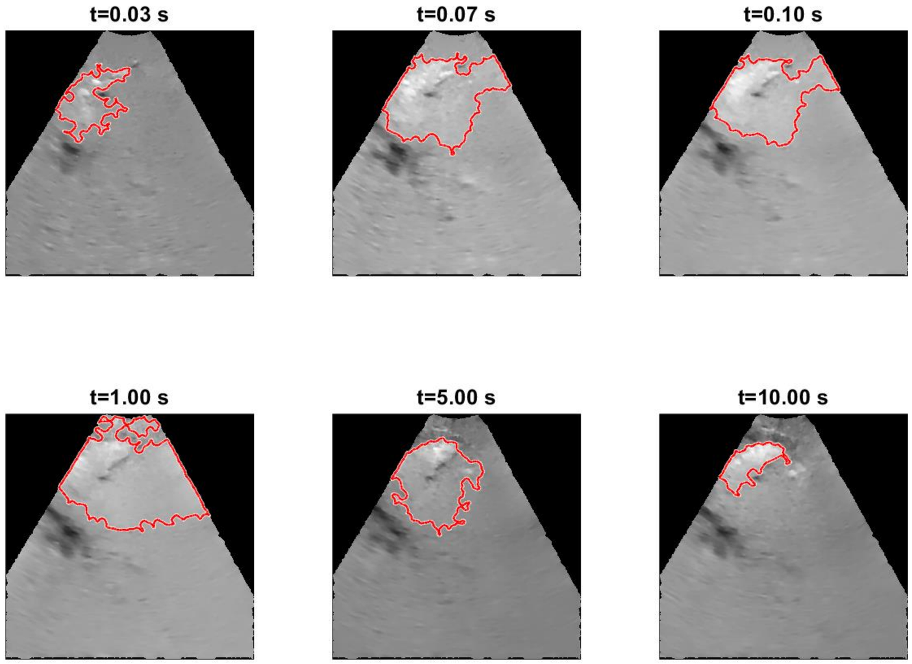

Figure S6: Selected frames of the filtered and segmented video for subject B11

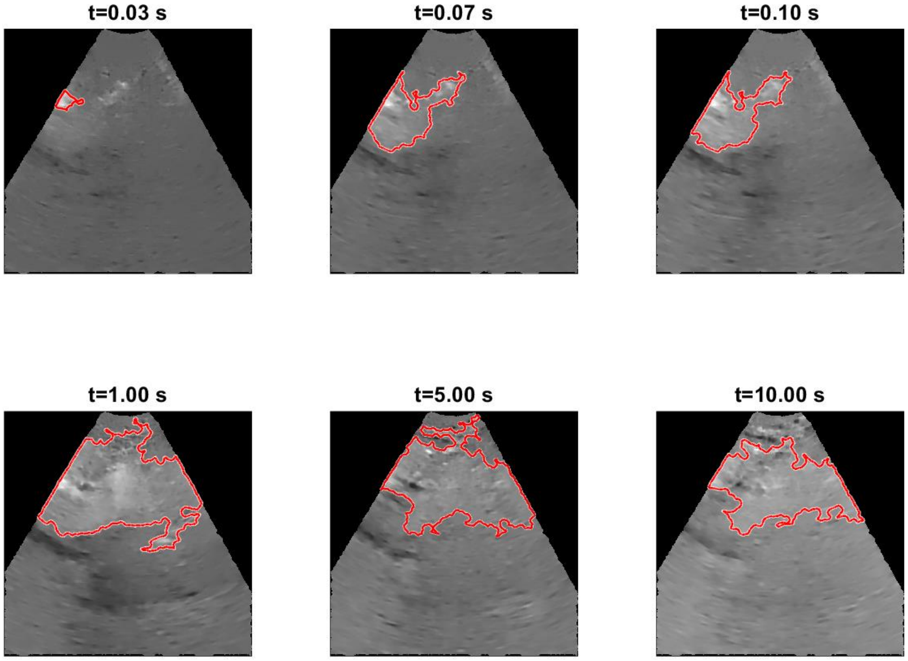

Figure S7: Selected frames of the filtered and segmented video for subject C11

References

1. Otsu N. A threshold selection method from gray-level histograms. IEEE transactions on systems, man, and cybernetics. 1979;9(1):62-66. Available

2. Paris S, Hasinoff SW and Kautz J. Local Laplacian filters: Edge-aware image processing with a Laplacian pyramid. ACM Trans Graph. 2011;30(4):68. Available
